# Supplementary material for: Combined abdominal heterotopic heart and aorta transplant model in mice
Source: PLoS One. 2020 Jun 22;15(6):e0230649. doi: 10.1371/journal.pone.0230649 (PMC7307752; doi:10.1371/journal.pone.0230649)
Supplement: Attachment — Submitted filename: PONE-D-20-06026_Response to Reviewers.docx [file pone.0230649.s002.docx]

# Response to the reviewers’ comments

## Reviewer #1

“Combined Abdominal Heterotopic Heart and Aorta Transplant Model in Mice” is a manuscript that presents a novel microsurgical technique intended to improve upon the heart and aorta/carotid graft. The presented technique improves on the existing one because it has shorter graft ischemic times and is centered in only one body cavity. This model and the existing model are important as it may be used to recapitulate the clinical process of aortic vasculopathy. The methodology of the surgery is meticulously explained, and the statistics are appropriately performed. The illustrations are of good quality and add to the overall work. The discussion content is well thought-out and provides context for the study. This paper is marred by poor grammar, and since PLOS one does not provide proofreading and copyediting support, I do not believe it is publishable in its current state. I also suggest the following minor revisions for this manuscript in order to consider it for publication:

**Response:** We thank the reviewer for their review and comments. We have thoroughly reviewed the text and significantly improved the grammar of the manuscript.

It would be helpful to the reader to provide the true distributions of the times for the operation (ischemic time, harvesting time), instead of reporting the approximate times. The authors should present the median time and the distribution (this could be represented graphically as well).

**Response:** We have included an additional figure highlighting (new Figure 6.) summarizing our mean times (including range) for donor heart harvesting, donor aorta harvesting, preparation of the recipient mouse, anastomosis of the heart and aorta (combined) and overall total ischemic time, and updated the text accordingly (see below).

**Figure 6:** Median time for donor heart harvest (red), donor aorta harvest (blue), transplant recipient preparation (green), total anastomosis time for the combined transplant procedure (pink) and total ischemic time (orange). Data are represented as median time (in minutes) ± the range in time for all procedures. n = 30 transplants.

The validation of the new surgical method should be more adequately described. The authors write, “we subsequently performed allografts using Balb/c donor aorta and hearts transplanted into C57Bl/6 recipients in the presence of double co-stimulatory blockade”. The number of replicates of this procedure should be detailed, and it should be noted that the histologic examination of the aorta and cardiac graphs (6a and 6b) are representative of these experiments, if that is in fact the case.

**Response:** We thank the reviewer for their valuable comments. We have added details of the number of replicates performed for allografts (n=3) and updated the figure legends for Figures 6 (new Figure 7) to reflect these are indeed representative micrographs.

Please add citations for the following sentence:

• Chronic allograft rejection remains the leading cause of graft loss one-year post-transplantation.

**Response:** We have added the following references to support the above statement:

1. Alba, A. C., E. Bain, N. Ng, M. Stein, K. O’Brien, F. Foroutan and H. Ross (2016). "Complications after heart transplantation: Hope for the best, but prepare for the worst." Int J Transplant Res Med **2**: 022.
2. Lund, L. H., K. K. Khush, W. S. Cherikh, S. Goldfarb, A. Y. Kucheryavaya, B. J. Levvey, B. Meiser, J. W. Rossano, D. C. Chambers, R. D. Yusen, J. Stehlik, H. International Society for and T. Lung (2017). "The Registry of the International Society for Heart and Lung Transplantation: Thirty-fourth Adult Heart Transplantation Report-2017; Focus Theme: Allograft ischemic time." J Heart Lung Transplant **36**(10): 1037-1046.

PLOS one does not provide any copyediting and proofreading service. There are multiple grammatical errors found in this manuscript. I believe that it is not publishable in its current state. I would suggest proofreading the article again. Examples of the many grammatical errors found throughout the paper are listed below:

**Response:** We thank the reviewer for their comment and have significantly improved the grammar and clarity of the manuscript.

• The result has further heightened the need for a parenchymal organ in combination with aorta transplantation in exploring clinic(al) relevance of CAV.

**Response:** This sentence has been rewritten.

• Our results demonstrate that this new model is characterized by the reliable reproducibility.
oShould this sentence include “the”?

**Response:** This sentence has been rewritten.

• After 1 minute for the systemic heparinization, an aortotomy of abdominal aorta is made to decompress the blood circulatory system,

othis should be an aortotomy of “the” abdominal aorta

**Response:** This sentence has been corrected.

• The IVC and the right superior vena cava (SVC) were proximally ligated with 7-0 sutures, respectively.
oIn this case respectively does not make sense.

**Response:** This sentence has been corrected.

## Reviewer #2

Dear authors,

Thank you for submitting your manuscript titled "Combined abdominal heterotopic heart and aorta transplant model in mice" to PLOS ONE. It was a very well written manuscript and I commend you on accomplishing this very difficult microsurgery. Your description of the operation, as well as the diagrams truly help the reader follow along the operation. I do have a couple questions :

1. Have you compared this technique to the "conventional" heterotopic heart transplantation in mice? As in, have you compared the histology, alloresponses, coronary inflammation, etc between the techniques. This technique seems more difficult, with more anastomoses. If everything is similar, is there a reason to do this technique?

**Response:** We have compared this novel technique to conventional “single” heterotopic heart transplantation model, in terms of heart allograft survival time and histology responses in the presence of double co-stimulatory blockade. Both models showed a long-term heart allograft (*Balb/c* to *C57Bl/6*) survival time (more than 90 days) and mild histology immune responses, due to a strong immunosuppressive effect of double co-stimulatory blockade. This has been mentioned in our article.

A limitation of this study is that we have not performed the single aorta transplantation in mice to compare changes in the transplanted aorta with that in our combined heart and aorta transplantation model. However, this work has been well performed by other groups (*Ensminger et al. 2002, Soleimani 2008*), and they demonstrated that acute rejection events in the transplanted heart is an important contributor to development of CAV in aorta grafts. Their findings indicate that a combined parenchymal organ transplant is necessary to develop clinically relevant CAV in an aortic graft. The aim of our present study is to develop a novel combined heart and aorta transplantation method in mice to simplify and refine current existing models.

In addition to a heterotopic heart transplantation, this novel technique also includes a segment of aortic transplantation, thus, making this combined transplantation procedure more difficult. However, a well-trained microsurgeon should be able to achieve a high success rate in this model with sufficient practice. The rationale for this novel model is as follows:

(1) In a single mouse heterotopic heart transplantation model, without immunosuppressive agents, fully MHC-mismatched cardiac allograft induce strong alloreactive T cell responses that mediate rapid graft destruction (*Sho et al*. 2002). Therefore, the study of CAV is not possible due to rapid heart graft failure.
(2) With a single mouse aorta transplantation model, the aortic allograft in the absence of immunosuppression can undergo long-term allograft survival, even across fully MHC-mismatched donor and recipient. Thus, the aortic graft is spared from acute rejection, which is normally observed in other solid organ transplant models. Acute rejection episodes have been considered as an important factor for the future development of CAV (*Azuma et al. 1994, Alba et al. 2016, Lund et al. 2017*). Therefore, the vascular changes in single aortic allograft transplantation model may not represent those that develop in solid-organ transplants (*Ensminger et al. 2000*).
(3) To address issues mentioned above, investigators have developed the combined heart and aorta/carotid artery transplantation model in mice to investigate the potential impact of acute rejection on CAV (*Ensminger et al. 2000, Soleimani et al. 2008*). Compared with isolated carotid allografts, a significantly greater degree of intimal hyperplasia of *C57Bl/10* carotid allografts was reported when transplanted in combination with a *Balb/c* heart graft into a *C3H* recipient, indicating that CAV is promoted by acute parenchymal rejection of the heart (*Soleimani et al. 2008*). This finding experimentally confirms that the acute parenchymal rejection is an important contributor to development of CAV, and further emphasizes that a combined model may provide more clinically relevant understanding of mechanisms that are involved in the development of CAV in an aorta/carotid artery graft.
(4) In current models of combined aorta/carotid and heart transplantation in mice, implantation of two individual grafts into two different surgical sites is utilized (abdominal cavity and cervical region, respectively). This approach has many limitations, including additional surgical procedures and prolonged anaesthesia, resulting in increased surgical complications and poor outcomes. To minimize the surgical insult to the recipient animal, the two transplantation procedures have to be performed on separate days (*Ensminger et al. 2000*), consequently increasing animal use.
(5) To address the issue above, we developed this simplified model, in which two individual grafts are transplanted into a single surgical site of abdomen with a high success rate. The advantages of this novel technique include the avoidance of unnecessary surgical trauma to animals, shortening of ischemic and overall operative times, and reducing animal usage (in the case of multiple survival surgeries, donor aorta and heart in other techniques need to be harvested from separate animals).
(6) The novel anastomotic technique adopted in our new model, sharing a common anastomotic stoma on the recipient’s infrarenal aorta with two individual graft vessels for revascularization of grafts, provides a new methodological option for the development of experimental multi-organ transplantations of animal models.
1. Ensminger, S. M., J. S. Billing, P. J. Morris and K. J. Wood (2000). "Development of a combined cardiac and aortic transplant model to investigate the development of transplant arteriosclerosis in the mouse." J Heart Lung Transplant **19**(11): 1039-1046.
2. Soleimani, B., F. Fu, P. Lake and V. C. Shi (2008). "Development of a combined heart and carotid artery transplant model to investigate the impact of acute rejection on cardiac allograft vasculopathy." J Heart Lung Transplant **27**(4): 450-456.
3. Sho, M., A. Yamada, N. Najafian, A. D. Salama, H. Harada, S. E. Sandner, A. Sanchez-Fueyo, X. X. Zheng, T. B. Strom and M. H. Sayegh (2002). "Physiological mechanisms of regulating alloimmunity: cytokines, CTLA-4, CD25+ cells, and the alloreactive T cell clone size." J Immunol **169**(7): 3744-3751
4. Azuma, H. and N. L. Tilney (1994). "Chronic graft rejection." Curr Opin Immunol **6**(5): 770-776.
5. Alba, A. C., E. Bain, N. Ng, M. Stein, K. O’Brien, F. Foroutan and H. Ross (2016). "Complications after heart transplantation: Hope for the best, but prepare for the worst." Int J Transplant Res Med **2**: 022.
6. Lund, L. H., K. K. Khush, W. S. Cherikh, S. Goldfarb, A. Y. Kucheryavaya, B. J. Levvey, B. Meiser, J. W. Rossano, D. C. Chambers, R. D. Yusen, J. Stehlik, H. International Society for and T. Lung (2017). "The Registry of the International Society for Heart and Lung Transplantation: Thirty-fourth Adult Heart Transplantation Report-2017; Focus Theme: Allograft ischemic time." J Heart Lung Transplant **36**(10): 1037-1046.

2. Unlike other solid organs, the heart is a dynamic structure that can fail with improper preload and afterload management which is critical post-transplantation. This can affect immunosuppression. Since this technique creates an organ in series, it may not be representative of the pressures seen in a real transplant. This may not matter in a liver for example. Do you think that since the heart is not exposed the similar pressures as in an orthotopic heart transplant that this technique may not be the best to address CAV? Please address. I think this is a major limitation of such models.

**Response:** We thank the reviewer for their valuable comment. An orthotopic heart transplantation model would undoubtedly be the best choice to address clinically relevant CAV. However, due to technical limitations, orthotopic heart transplantation in rodents is currently not possible (*Plenter et al*. 2014, *Figueiredo et al. 2009*). To date, the heterotopic heart transplantation in mice is one of the most frequently used transplant models since it was first described in 1973 (*Corry et al*. 1973), although there are, with all models, certain limitations. After grafting, the heterotopically transplanted heart quickly starts contracting, which leads to a retrograde blood flow from the recipient abdominal aorta via the donor-ascending aorta directly into the coronary arteries, thus irrigating the myocardium via the capillary network. Blood flow is then drained from coronary circulation back into the coronary veins, coronary sinus, the right atrium (RA) and the right ventricle (RV), and finally reaches the recipient IVC via the main pulmonary trunk. Hence, the model is associated with a “non-functional” heart graft because it does not pump blood to maintaining physiological blood pressure despite heart graft contractions. The retrograde perfusion in the beating but “non-functional” graft, with a total mechanical unloading of left ventricle (LV), is one of the major shortcomings of the model, which leads to mural thrombi formation in the LV and progressive atrophy of the transplanted graft (*Baldwin et al.* 2014). To address this issue, Figueiredo et al. (*Figueiredo et al.* 2009) established the partial LV loading model in mice by heterotopic combined heart/lung transplantation. However, whether the volume-loaded heterotopic heart transplantation is superior to the classical myocardial unloading model in rodents remains controversial (*Didié et al*. 2013; *Tang-Quan et al.* 2010; *Liu et al*. 2015). Nevertheless, based on advantages of the immediate vascularization and the ease of monitoring rejection non-invasively, the heterotopic heart transplantation model in mice still remains the cornerstone of basic organ transplant and immunology research while kidney, lung, liver and pancreas transplants are available in mice (*Plenter et al*. 2014).

1. Baldwin, W. M., 3rd, C. A. Su, T. M. Shroka and R. L. Fairchild (2014). "Experimental models of cardiac transplantation: design determines relevance." Curr Opin Organ Transplant **19**(5): 525-530.
2. Corry, R. J., H. J. Winn and P. S. Russell (1973). "Primarily vascularized allografts of hearts in mice. The role of H-2D, H-2K, and non-H-2 antigens in rejection." Transplantation **16**(4): 343-350.
3. Didie, M., D. Biermann, R. Buchert, A. Hess, K. Wittkopper, P. Christalla, S. Doker, F. Jebran, F. Schondube, H. Reichenspurner, A. El-Armouche and W. H. Zimmermann (2013). "Preservation of left ventricular function and morphology in volume-loaded versus volume-unloaded heterotopic heart transplants." Am J Physiol Heart Circ Physiol **305**(4): H533-541.
4. Figueiredo, J. L., M. Nahrendorf, D. E. Sosnovik and R. Weissleder (2009). "MRI of a novel murine working heart transplant model." Circ Heart Fail **2**(3): 272-274.
5. Liu, Y., P. Maureira, G. Gauchotte, A. Falanga, V. Marie, A. Olivier, F. Groubatch, C. Gu, P. Y. Marie and N. Tran (2015). "Effect of chronic left ventricular unloading on myocardial remodeling: Multimodal assessment of two heterotopic heart transplantation techniques." J Heart Lung Transplant **34**(4): 594-603.
6. Plenter, R. J. and T. J. Grazia (2014). "Murine heterotopic heart transplant technique." J Vis Exp(89).
7. Tang-Quan, K. R., J. Bartos, T. Deuse, E. Churchill, H. Schafer, H. Reichenspurner, D. Mochly-Rosen, R. C. Robbins and S. Schrepfer (2010). "Non-volume-loaded heart provides a more relevant heterotopic transplantation model." Transpl Immunol **23**(1-2): 65-70.

3. Despite heparin, there could still be issues with thrombosis. How can you tell if the coronaries are patent in the transplanted heart? Is the mouse continued on anticoagulation?

**Response:** Anticoagulation with heparin is only administrated to the donor animal during the heart and aorta graft harvesting procedure. Due to small size of blood vessels in mice, vascular anastomoses are technically very difficult. Normally, thrombosis is directly induced by poor surgical technique for anastomosis, and it cannot be prevented with anticoagulation. Therefore, after heart transplantation, the recipient animal is not subject to any anticoagulation therapy. After grafting, one can determine if the coronaries in the transplanted heart are patent by observing whether the heart graft immediately fills with blood. If this is the case, it will become bright red in color and sinus rhythm will start within 1 min (after a short episode of fibrillation).

4. Do the transplant hearts go into arrhythmia?

**Response:** Arrhythmia frequently occurs in patients with orthotopic heart transplantation. Due to ischemia/reperfusion injury resulting in sinus node dysfunction, 2-24% of patients require permanent pacemaker implantation after orthotopic heart transplantation (*Mallidi et al*. 2017). In the mouse heterotopic heart transplantation model, after grafting, the transplanted heart graft quickly starts contracting with a sinus rhythm. The graft function is assessed by trans-abdominal palpation of the cardiac contractions daily. However, to our knowledge, the incidence of arrhythmia has not been reported in heterotopic heart transplantation in mice, likely due to the “non-functional” status of the heart graft (see response #2). This abnormality may be only detectable with electrocardiography (ECG) study.

1. Mallidi, H. R. and M. Bates (2017). "Pacemaker Use Following Heart Transplantation." Ochsner J **17**(1): 20-24.

5. Please review grammar and syntax.

**Response:** We thank the reviewer for their comment and have significantly improved the grammar and syntax of the manuscript.

Thank you very much for a very interesting manuscript.

**Response:** We appreciate your time and efforts in reviewing our manuscript and thank you for your valuable feedback and comments about our manuscript.
