# Supplementary material for: Combined abdominal heterotopic heart and aorta transplant model in mice
Source: PLoS One. 2020 Jun 22;15(6):e0230649. doi: 10.1371/journal.pone.0230649 (PMC7307752; doi:10.1371/journal.pone.0230649)
Supplement: Attachment — Submitted filename: PONE-D-20-06026R2_Response to Reviewers.docx [file pone.0230649.s003.docx]

# Response to the reviewer’s comments

At the end of the first paragraph of the results the following sentence is hard to understand: “Survival of both the syngeneic aorta and heart graft was greater than 90 days.” Is this referring to all of the mice that survived the procedure?

**Response:** The highlighted sentence indeed refers to all of the syngrafts that survived the surgical procedure (29 of the 30 performed in this group). We have revised the sentence in the manuscript to clarify this detail as follows:

“The remaining 29 of 30 syngrafts survived greater than 90 days with maintained transplanted heart function (as assessed by palpation).”

I do not believe that the graft survival should be presented as 6/6 in the second paragraph of the results (since you are referring to three cardiac graphs and three aortic graphs). I think it should be presented as 3/3 cardiac graphs and 3/3 aortic graphs.

**Response:** The previous sentence was meant to refer to 6 of 6 total transplants for this experimental group (three *Balb/c* to *C57Bl/6* allografts receiving double co-stimulatory blockade and three *C57Bl/6* to *C57Bl/6* syngrafts). We have revised the sentence in the manuscript to clarify this detail as follows:

“For this group, 3/3 allografts and 3/3 syngrafts survived for more than 90 days, with maintained transplanted heart function (as assessed by palpation.”
